# Hereditary Xerocytosis due to Mutations in* PIEZO1* Gene Associated with Heterozygous Pyruvate Kinase Deficiency and Beta-Thalassemia Trait in Two Unrelated Families

**DOI:** 10.1155/2017/2769570

**Published:** 2017-03-06

**Authors:** Elisa Fermo, Cristina Vercellati, Anna Paola Marcello, Anna Zaninoni, Richard van Wijk, Nadia Mirra, Cristina Curcio, Agostino Cortelezzi, Alberto Zanella, Wilma Barcellini, Paola Bianchi

**Affiliations:** ^1^U.O.C. Oncoematologia, U.O.S. Fisiopatologia delle Anemie, Fondazione IRCCS Ca' Granda Ospedale Maggiore Policlinico, Milano, Italy; ^2^Department of Clinical Chemistry and Haematology, University Medical Center Utrecht, Utrecht, Netherlands; ^3^U.O.C. Pronto Soccorso, Pediatria Ambulatoriale e DH/MAC, Fondazione IRCCS Ca' Granda Ospedale Maggiore Policlinico, Milano, Italy; ^4^U.O.S.D. Genetica Medica, Fondazione IRCCS Ca' Granda Ospedale Maggiore Policlinico, Milano, Italy; ^5^Universita degli Studi di Milano, Milano, Italy

## Abstract

Hereditary xerocytosis (HX) is a rare disorder caused by defects of RBC permeability, associated with haemolytic anaemia of variable degree and iron overload. It is sometimes misdiagnosed as hereditary spherocytosis or other congenital haemolytic anaemia. Splenectomy is contraindicated due to increased risk of thromboembolic complications. We report the clinical, haematological, and molecular characteristics of four patients from two unrelated Italian families affected by HX, associated with beta-thalassemia trait and heterozygous pyruvate kinase deficiency, respectively. Two patients had been splenectomised and displayed thrombotic episodes. All patients had iron overload in the absence of transfusion, two of them requiring iron chelation. The diagnosis of HX was confirmed by LoRRca Osmoscan analysis showing a left-shifted curve.* PIEZO1* gene sequencing revealed the presence of mutation p.E2496ELE, showing that this is one of the most frequent mutations in this disease. The concomitant defects did not aggravate the clinical phenotype; however, in one patient, the initial diagnosis of pyruvate kinase deficiency delayed the correct diagnosis of HX for many years and resulted in splenectomy followed by thrombotic complications. The study underlines the importance of a precise diagnosis in HX, particularly in view of splenectomy, and the need of a molecular confirmation of suspected RBC enzymopathy.

## 1. Introduction

Dehydrated stomatocytosis, also known as hereditary xerocytosis (HX, OMIM 194380), is the most frequent variant of hereditary stomatocytoses, a group of rare disorders characterized by a leak of monovalent cations (Na^+^ and K^+^) from the red blood cells (RBCs) [1, 2]. In particular, in HX red cells a decreased intraerythrocytic K^+^ and total cations content is not accompanied by a proportional net gain of sodium and water, ultimately resulting in erythrocyte dehydration [3]. HX patients usually present with mild to moderate chronic haemolytic anaemia and splenomegaly, increased reticulocyte count, and slight macrocytosis; moreover, erythrocyte mean corpuscular haemoglobin concentration (MCHC) is increased and erythrocyte osmotic fragility is decreased. Blood smears show variable numbers of stomatocytes, but usually less than 10% [1]; HX is characterized by a propensity to iron overload [4]. The majority of HX cases has a mild presentation and may be overlooked for many years or sometimes misdiagnosed as hereditary spherocytosis or other congenital haemolytic anaemia [1]. However, unlike hereditary spherocytosis in which it is highly beneficial, splenectomy is contraindicated in HX due to increased risk of thromboembolic complications [5–7]. 

Osmotic gradient ektacytometry, which measures red cell deformability, osmotic fragility, and cell hydration, is the gold standard test for the definitive diagnosis and typically shows a leftward shift of the bell-shaped curve [1, 8]. Sixteen different mutations in* PIEZO1* gene have been identified in 27 unrelated families with HX, primarily located in the highly conserved COOH terminus of the protein [9–18]. Functional studies of HX-associated* PIEZO1* mutations demonstrate a partial gain-of-function phenotype with many mutants demonstrating delayed inactivation [10, 11, 19], suggesting increased cation permeability that leads to HX erythrocyte dehydration.

In this paper we report the clinical, haematological, and molecular characterization of two unrelated Italian families with HX due to mutation p.E2496ELE in* PIEZO1* gene associated with beta-thalassemia trait in one and heterozygous pyruvate kinase (PK) deficiency in the other. By reviewing the other cases reported in literature carrying the same mutation, we exclude a synergetic effect between HX and the concomitant disorders. In one patient the initial diagnosis of pyruvate kinase deficient haemolytic anaemia delayed the correct diagnosis of HX and resulted in splenectomy followed by thrombotic complications.

## 2. Case Report

### 2.1. Family A

**Figure 1:**
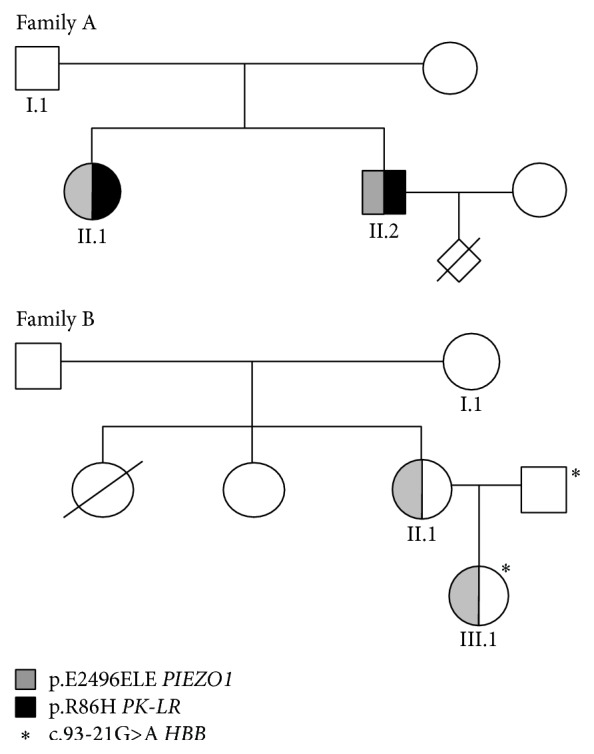
Genealogical trees of the reported families.

Two siblings, born after uncomplicated pregnancy form unrelated parents, were studied (Figure 1); the father (AI.1) was reported to have chronic haemolytic anaemia of unknown origin. AII.1 displayed chronic haemolysis and jaundice since infancy; she was diagnosed with PK deficiency on the basis of decreased enzyme activity and splenectomised in 1979 at the age of 21. After splenectomy jaundice disappeared and Hb levels increased from 9.4 to 11 g/dL, two episodes of deep vein thrombosis (DVT) occurred, at the age of 26 and 54. Hyperferritinemia (1162 ng/ml) was documented at the age of 46 and one cycle of deferoxamine chelation was done for 3 months only because of intolerance; at the age of 55 T2^*∗*^ MRI revealed marked liver iron overload (*R* = 0.51) and patient started deferasirox treatment.

AII.2 was first studied in 1992 at the age of 29 after his wife's first pregnancy interruption at 26 weeks of gestation for severe hydrops foetalis attributed to parvovirus B19 infection. On that occasion, Hb levels were 12.5 g/dL, reticulocytes were 316 × 10^9^/L, unconjugated bilirubin was 5.9 mg/dL, and serum ferritin was 723 ng/ml; PK activity was slightly decreased (10.2 IU/gHb, normal values 13.3 ± 2.29); however, because of family history he was diagnosed with PK deficiency. Physical examination revealed scleral jaundice and splenomegaly. He underwent cholecystectomy at the age of 30. Both siblings were referred to our Centre for reevaluation of haemolytic anaemia and iron overload. The patients' clinical and haematological data at the time of this study are shown in Table 1.

**Figure 2:**
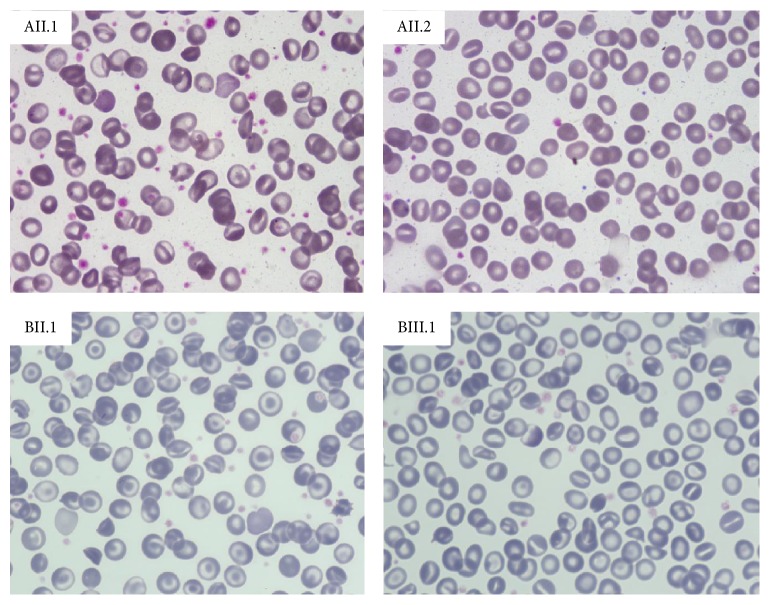
Light microscopy of peripheral blood, 63x.

Both patients AII.1 and AII.2 displayed mild macrocytic anaemia and reticulocytosis, 13–15% stomatocytes in peripheral blood smear examination with 7% target cells, and 2–4% schistocytes, ovalocytes, and spherocytes (Figure 2). Increased serum ferritin levels were also detected in the absence of blood transfusions;* HFE* genotype was H63D/wt. Interestingly AII.1 showed mild spectrin deficiency with normal EMA binding test. RBC PK activity was decreased in both siblings (7 IU/gHb and 10.8 IU/gHb, resp.). Direct sequencing of the entire coding region, intronic flanking regions, and promoter of* PK-LR* gene showed heterozygosity for the c.257G>A mutation (p.R86H). Because PK deficiency is an autosomal recessive disease this finding allowed excluding the previous diagnosis of PK deficiency and prompted us to investigate other causes of haemolysis in this family; the RBC morphology, the apparent dominant transmission, and the history of DVT in the splenectomised patient were suggestive of hereditary stomatocytosis.

**Figure 3:**
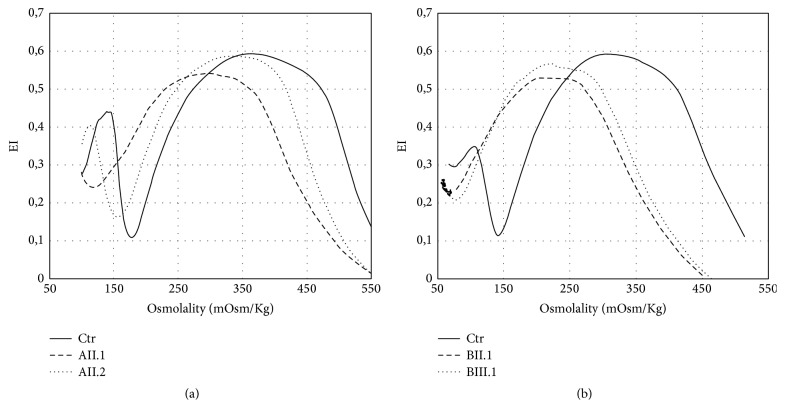
Results of LoRRca Osmoscan analysis in the affected patients compared to a normal control performed by Laser-Assisted Optical Rotation Cell Analyzer (LoRRca MaxSis, Mechatronics, Hoorn, The Netherlands). The osmotic gradient curves reflect RBC deformability as a continuous function of suspending medium osmolality. The following parameters were evaluated: EImax (maximal deformability, reflecting mean cellular surface area), Omin (osmolality at which deformability reaches its minimum, reflecting mean cellular surface-to-volume ratio), and Ohyper (the osmolality in the hypertonic region corresponding to 50% of the EImax, reflecting mean cellular hydration status).

LoRRca Osmoscan (Figure 3(a)) showed in AII.2 a left shift of the curve (Omin 156, n.v. 151–175; Ohyper 456, n.v. 473–534), indicating the presence of dehydrated red cells. In AII.1, a more pronounced left shift (Omin 119, Ohyper 428), was also associated with decreased EImax (0.541, n.v. 0.586–0.610), in line with the spectrin deficiency detected by SDS-PAGE analysis.* PIEZO1 *gene sequencing revealed in both siblings heterozygosity for the mutation c.7479_7484dupGGAGCT in exon 51 (p.E2496ELE), thus confirming the diagnosis of HX.

### 2.2. Family B

Patient BII.1 was first recognized to be anaemic at the age of 15 years because of scleral jaundice; at the age of 23 in 1995 she was diagnosed with hereditary stomatocytosis based on the presence of stomatocytes at peripheral blood smear examination (12%); in that occasion, she had Hb 11.9 g/dL, MCV 95 fL, reticulocytes 550 × 10^9^/L, serum ferritin 421 ng/ml, transferrin saturation 70%, and unconjugated bilirubin 6.7 mg/dL. Abdominal ultrasound showed splenomegaly (18 × 12 cm), hepatomegaly, and gallstones. Family study revealed in the mother (BI.1) a compensated haemolysis (Hb 12.6 g/dL, reticulocytes 311 × 10^9^/L, and unconjugated bilirubin 2 mg/dL) with 11% stomatocytes, suggesting dominant transmission of the disease.

One year later the patient underwent cholecystectomy for gallstones, associated with splenectomy. Surgery had no effect on Hb levels; postsplenectomy reticulocytes were 200–300 × 10^9^/L and serum ferritin was 300–400 ng/ml. At the age of 30 years, the patient became pregnant and she gave birth to a girl (BIII.1); pregnancy was complicated by gestosis, with delivery at 35 weeks. At birth the child was admitted to the Intensive Care Unit for neonatal immaturity and respiratory distress, and she was diagnosed with hereditary stomatocytosis associated with beta-thalassemia trait (Hb 10.6 g/dL, MCV 71.9 fL, stomatocytes 22%, reticulocytes 260 × 10^9^/L, and unconjugated bilirubin 1.09 mg/dL).

The mother and daughter were referred to our hospital for reevaluation of their anaemia and to confirm the diagnosis at molecular level. At the last follow-up patient BII.1 reported that a thromboembolic event occurred 20 years after splenectomy. Patient BII.1 displayed compensated haemolytic anaemia with 22% stomatocytes at peripheral blood smear examination, 13% target cells, and 2% schistocytes (Table 1 and Figure 2). Ferritin levels and transferrin saturation were increased in the presence of a normal* HFE* genotype. Bone marrow evaluation showed marked dyserythropoiesis. Case BIII.1 had mild microcytic anaemia, reticulocytosis, and increased ferritin levels; 17% of stomatocytes were detected at blood smear examination (Table 1 and Figure 2); the molecular testing for* HBB* gene revealed the presence of the splicing mutation c.93-21G>A at heterozygote level, confirming the beta-thalassemia trait.

LoRRca Osmoscan (Figure 3(b)) showed the pronounced left shift of the curve typical of dehydrated stomatocytosis (Omin 71 and 76, resp., n.v. 136–151; Ohyper 342 and 352, n.v. 447–474).* PIEZO1 *gene sequencing revealed in both cases the presence of the mutation p.E2496ELE.

## 3. Discussion

There is an increasing interest in defects of RBC permeability due to the recent advances in knowledge of their molecular bases. Considered to be a group of very rare diseases, the number of reported cases of HX in the past three years is increased after the identification of the causative genes, such as* PIEZO1* [9–11] and, more recently,* KCNN4 *codifying for Gardos channel [20–23].

The wide heterogeneity of clinical presentation and in particular the occurrence of mild/compensated cases may contribute to delay of the diagnosis [14, 16]. Moreover, some patients displaying very few stomatocytes at the blood film examination may be misdiagnosed as hereditary spherocytosis or as suffering from an enzyme deficiency [1]. This was the case in patient AII.1, initially diagnosed as PK deficiency based on decreased enzyme activity, who underwent splenectomy and was eventually identified as HX with 30-year delay: the apparently dominant transmission of the anaemia, the absence of the expected increase of reticulocytes number after splenectomy, and* PK-LR* genotyping showing monoallelic mutation ruled out the diagnosis of PK deficiency as a primary cause of haemolysis. This case therefore pinpoints the need of confirming a suspected RBC enzymopathy at the molecular level.

More than in other haemolytic anaemia, a precise diagnosis is of the utmost importance in HX in light of the increased risk of thrombotic complications following splenectomy [5–7] reported in all the splenectomised patients with* PIEZO1* mutations but one. Patient AII.1 displayed two DVT episodes 5 and 33 years after splenectomy, and patient BII.1 had pulmonary embolism 20 years after surgery. It is worth mentioning that the only patient reported in the literature without thromboembolic complication after splenectomy had a relatively short follow-up [17].

Although usually well compensated and requiring no blood transfusions, dehydrated stomatocytosis is a heavily iron loading condition [4, 14], as also shown in our cases. The causes of this are not well established: haemolysis is commonly mild and it is unlikely to be per se the main cause of iron overload. An important factor in this regard may be dyserythropoiesis, which was also present in patient BII.1. The occurrence of dyserythropoiesis has been documented in a variant of hereditary stomatocytosis due to Gly796Arg mutation of the erythroid anion exchanger [24] and in one case with HX associated with hereditary high phosphatidylcholine haemolytic anaemia [17] but excluded in another* PIEZO1* mutated patient described by Archer et al. [14].

HX has been long known to be a pleiotropic syndrome which combines in some families with haemolytic anaemia and perinatal edema; the latter, usually transient and of relatively benign prognosis [25–28], has been recently correlated with* PIEZO1* mutation by Andolfo et al. [10]. The nonimmune foetal hydrops recorded in family A is the most severe form so far described in patients with* PIEZO1* mutations.

LoRRca Osmoscan proved to be very useful in orienting the diagnosis toward HX, later confirmed by the* PIEZO1* gene analysis. Interestingly, patient AII.1 also displayed a decrease in EImax, as found in the case reported by Grootenboer-Mignot et al. [27]. In our patient the decrease in EImax is in line with the SDS-PAGE finding of a moderate spectrin deficiency. Spectrin deficiency in the absence of any mutation in RBC cytoskeletal proteins documented by whole exome sequencing has been reported in hereditary stomatocytosis due to* KCNN4* mutation [23] and considered to be a secondary effect of a membrane perturbation [29, 30] disclosed by splenectomy [31].

All the four patients here described were heterozygous for mutation p.E2496ELE. The mutation was already reported in 8 unrelated cases described by Albuisson et al. [11] and very recently in a Japanese family [17] (Table 2). Although being also detected in 2 of 600 healthy French controls, this mutation was considered pathogenic; in fact, patch-clamp experiments in transfected HEK293 cells demonstrated a considerable increase in the inactivation time in the mutant compared to wild-type channel kinetics, indicating that it represents a gain-of-function mutation [11]. The finding of p.E2496ELE mutation also in our unrelated families (the first two families of Italian origin characterized at molecular level) supports its pathogenicity and confirms that this is one of the most frequent mutations in HX, with 15 patients so far reported in literature.

Combined defects of red cell membrane and/or metabolism are very rare, and the fact that carriership for a metabolic defect may be a modifier for the clinical expression of a membrane defect is still debated [32, 33]; in particular, the copresence of beta-thalassemia trait seems to reduce the degree of haemolysis in patients with hereditary spherocytosis [34–37]. In the presented cases the clinical phenotype does not differ from the HX cases reported in literature (Table 2), thus excluding a synergetic effect between HX and the concomitant disorders.

In conclusion, our data underline the importance of a precise diagnosis in HX, particularly in view of splenectomy because of the increased thrombotic risk, and the need of a molecular confirmation of a suspected RBC enzymopathy.

## Figures and Tables

**Table 1:** Clinical and haematological data of the patients at the time of the study.

	AII.1_ _^#^	AII.2	BII.1_ _^#^	BIII.1	Normal values
Sex	F	M	F	F	
Age	55	50	39	9	
Detection of anaemia	Infancy	29 yrs	15 yrs	Birth	
Splenectomy (age)	Yes (21)	No	Yes (24)	No	
Cholecystectomy (age)	No	Yes (30)	Yes (24)	No	
Transfusions	No	No	No	No	
Hb (g/dL)	11	13	12	10.8	F 12–16; M 13.5–17.5
MCV (fl)	108	103	100	66.4^*∗*^	78–99
MCH (pg)	39.7	39.2	38.1	24.1	25–35
MCHC (g/dL)	36.5	37.8	38	36.2	31–37
Reticulocytes (×10^9^/L)	166	273	198	423	24–84
Stomatocytes (%)	13	15	33	22	
PLTs (×10^9^/L)	696	172	875	329	130–400
WBCs (×10^9^/L)	8.4	7.68	9.31	8.15	4.8–10.8
Unconj bilirubin (mg/dL)	1.33	4.82	1.33	2.8	<0.8
Serum iron (*μ*g/dL)	101	150	167	n.a.	59–158
Serum ferritin (ng/mL)	745	1571	356	321	F 15–150; M 30–400
Transferrin (*μ*g/dL)	149	170	237	221	200–360
Transferrin saturation (%)	56	71	75	n.a.	16–54
GLT (sec)	180	68	480	195	23–45
AGLT (sec)	>900	>900	>900	>900	>900
NaCl osmotic fragility	Reduced	Reduced	Reduced	Reduced	
Pink test (%)	27	28	16	16	11–33
EMA binding test	Normal	Normal	n.a.	n.a.	
Sp/Bd3 ratio	0.90	1.14	1.11	1.05	0.97–1.19
*HFE* genotype	H63D/wt	H63D/wt	wt/wt	n.a.	wt/wt
Gilbert genotype	6/6	6/6	6/7	n.a.	6/6
PK activity (IU/gHb)	7	10.8	nd	nd	11.9–16.1
2,3 DPG (nmol/gHb)	n.a.	14183	nd	nd	10540 ± 1720
ATP	n.a.	5674	nd	nd	4231 ± 630
*PK-LR* genotype	c.257G>A/wt	c.257G>A/wt	nd	nd	

^*∗*^ *β*-Thalassemia trait; n.a. = not available; nd = not determined.

^#^Postsplenectomy.

**Table 2:** Patients reported in literature with mutation p.E2496ELE in *PIEZO1* gene.

Reference	Case	Age at diagnosis	Family history	Perinatal edema	Splenectomy (age)	Thrombotic events	Stomatocytes	Typical HX ektacytometry	Hb (g/dL)	MCV (fL)	MCHC (%)	Retics (10^9^/L)	Ferritin (ng/mL)	HFE
[11]	K1	16	No	No	n.a.	n.a.	n.a.	Yes	10.3	102.8	32.5	136	n.a.	n.a.
[11]	K2	15	n.a.	n.a.	n.a.	n.a.	n.a.	Yes	14.4	98.5	36	202	n.a.	n.a.
[11]	K3	11	Yes	n.a.	n.a.	n.a.	n.a.	Yes	11.3	91.9	37.3	275	n.a.	n.a.
[11]	K5	21	Yes	No	n.a.	n.a.	n.a.	Yes	12.7	98.9	37.5	256	n.a.	n.a.
[11]	K6	18	Yes	No	n.a.	n.a.	n.a.	Yes	12.5	102.5	36	378	n.a.	n.a.
[11]	K7	42	Yes	n.a.	n.a.	n.a.	n.a.	Yes	12.6	108.6	36.5	290	n.a.	n.a.
[11]	F2	30	Yes	Yes	No	—	n.a.	Yes	9.4	99.1	n.a.	182	n.a.	n.a.
[11]	F3	26	Yes	No	Yes (27)	Yes	5%	Yes	13.9	120	33.8	220	2910	n.a.
[17]	Daughter_ _^#^	50	Yes	No	Yes (38)	No	Yes	n.a.	7.9	133.9	n.a.	3.9%	4315	wt
[17]	Son_ _^#^	41	Yes	No	No	—	Yes	n.a.	6.6	101.1	n.a.	4.9%	4350	wt
This study	AII.1^$^	55	Yes	No	Yes (21)	Yes	13%	Yes	11	108	36.5	166	939	H63D/wt
This study	AII.2^$^	50	Yes	No	No	—	15%	Yes	13	103	37.8	273	1571	H63D/wt
This study	BII.1	23	Yes	No	Yes (23)	No	33%	Yes	12	100	38	198	356	wt
This study	BIII.1^*∗*^	Birth	Yes	No	No	—	22%	Yes	10.8	66.4	36.2	423	321	n.a.

__^*∗*^HX associated with *β*-thalassemia trait; _ _^$^HX associated with heterozygote PK deficiency.

__^#^HX associated with hereditary high phosphatidylcholine haemolytic anaemia (HHPCHA).

n.a. = not available.
